# Supplementary material for: Visible-light-driven amino acids production from biomass-based feedstocks over ultrathin CdS nanosheets
Source: Nat Commun. 2020 Sep 29;11:4899. doi: 10.1038/s41467-020-18532-3 (PMC7525434; doi:10.1038/s41467-020-18532-3)
Supplement: Supplementary file 1 — Supplementary Information [file 41467_2020_18532_MOESM1_ESM.pdf]

Supplementary Information

Visible-Light-Driven Amino Acids Production from Biomass-based Feedstocks over Ultrathin CdS Nanosheets

Song et al.

Supplementary Methods

Commercial materials and chemicals: TiO_2, ZnO, and Commercial CdS were purchased from Sigma-Aldrich and used directly. $Cd(OAc)_2 \cdot 2H_2O$ (98 %), $Cd(NO_3)_2 \cdot 4H_2O$ (98 %), $Bi(NO_3)_3 \cdot 5H_2O$ (≥ 98 %), $SC(NH_2)_2$ (≥ 99%), ethylenediamine (99 %), urea (99 %), ammonium metavanadate (≥ 99 %), L-cysteine (≥ 98 %), $CuCl_2$ (99 %), lactic acid (> 85 %), 3-phenyllactic acid (99 %), 2-hydroxyl butyric acid (98 %), 3-methylbutyric acid (99%), α-hydroxyisoproic acid (99 %), 2-hydroxy succinic acid (99 %), glycolic acid (99 %), mandelic acid (99 %), α-hydroxyl isobutyric acid (99 %), 3-hydroxybutyric acid (95 %) and 3-hydroxypropionic acid (30 % solution in water), pyruvic acid (98 %) and L-alanine (98 %) were purchased from Sigma-Aldrich. Ammonia solution (25 wt%) was purchased from VWR. All the chemicals were used as received.

Synthesis of *g*-C_3N_4: 10 g urea was grounded in a mortar for 0.5 h. After that, the samples were transferred to a crucible with cover and sealed with aluminum foil. The crucible was then heated from room temperature to 500 °C (10 °C/min) in furnace for 2 h in air.[1]

Synthesis of $BiVO_4$: 12.0 mmol of Ammonium metavanadate and $Bi(NO_3)_3 \cdot 5H_2O$ were separately dissolved in nitric acid solutions (50 mL; 2.0 M). And then the two solutions were mixed and formed a yellow homogeneous solution. After that, the mixture was transferred to a stainless-steel autoclave, heated at 453 K for 48 h.[2]

Synthesis of CuS: 0.03 g of L-cysteine was dispersed in the 15 mL of water, and then 0.125 mL of 1 M $CuCl_2$ aqueous solution was dropped into the above solution with stirring. The mixture was then transferred into a Teflon-line stainless steel autoclave, and kept at 160 °C for 12 h. The products were filtered and washed with water for several times, and dried at vacuum oven.[3]

Catalyst characterization: The morphology and microstructures of CdS catalysts were observed by using a transmission electron microscope (TEM) (FEI Tecnai 30) with 300 kV acceleration voltage. The crystal phase of CdS was analyzed by the X-ray diffraction (XRD) (X'Pert-Pro MPD), high-resolution TEM (HRTEM) and selected area electron diffraction (SAED). And the X-ray photoelectron spectroscopy (XPS) (ESCALAB 250Xi) was used to analyze the composition. The surface area and pore size distribution were measured on an ASAP 2020 Brunauer-Emmett-Teller (BET) analyzer. Varian-Cary 5000 spectrophotometer was used to record the diffuse reflectance Ultraviolet-visible (UV-vis) spectroscopic of photocatalysts. Inductively Coupled Plasma (ICP) tests were carried out on a Thermo Scientific iCAP 6000 series ICP spectrometer. The photoelectrochemical measurements of these photocatalysts were recorded on a CHI 660B electrochemical system. The working electrode

was prepared by cleaning an indium-tin oxide glass (ITO glass, 1 cm × 3 cm). In addition, the Pt plate served as the counter electrode, and the saturated calomel electrode served as the reference electrode. A 0.1 M Na_2SO_4 aqueous solution was used as the electrolyte and a 300 W Xenon lamp served as the light source. And the detailed process of preparing the working electrode is as follows, 10 mg of the photocatalysts were dispersed in 5 mL deionized water, and 1.5 mL homogeneous suspension was added dropwise directly onto the surface of ITO glass. Finally, the ITO glass with samples was dried at 80 °C for 1 h. Samples for electron spin-resonance (ESR) spectroscopy were performed at room temperature using a JES-X320 spectrometer, and 5, 5-dimethyl-l-pyrroline-N-oxide (DMPO, 50 mM) solution was used as paramagnetic species spin-trap agent. For in situ ESR measurements, 20 mmol lactic acid was added into oxygen-depleted ultra-pure water (20 or 16 mL), ammonia (0 or 4 mL) and 120 μL DMPO, then 10 mg CdS nanosheets or nanorods were dispersed in the above mixture solution (200 μL), by ultrasonic treatment. Then, the sealed glass capillary contained the above suspension was placed in a glass tube. The glass tube was then inserted into the ESR cavity and was irradiated under a 300 W Xenon lamp, and the spectra were recorded at selected times.

H_2 production experiment: LabSolar-3AG (PerfectLight Beijing) photocatalytic system was used to conduct H_2 production experiments. 10 mg of CdS was dispersed into the mixture solution (20 mmol lactic acid as the hole sacrificial agent and 18 mL water), and then sonicated for 0.5 h. Before reaction, the mixed solution was degassed thoroughly to remove air. A 300 W Xe lamp with UV cutoff filter (420-780 nm) was used as the light source and the online gas chromatograph (GC D7900P, TCD detector, N_2 carrier, 5Å molecular sieve column, Shanghai Fechcomp) was used to quantify the H_2-production rate.

Synthesis of d-labeled lactic acid: 20 mmol pyruvic acid was dissolved into 2 mL D_2O. 0.25 g commercial Pt/C was used as catalyst directly without pre-reduction. 35 bar D_2 was pressured into the reactor. The reaction temperature was 100 ^oC. Reaction time was 5 h. The pressure will decrease into 10 bar. Then, filtered the catalyst, add another 0.25 g Pt/C catalyst, 35 bar D_2 was pressured into the reactor again. The reaction temperature was 100 ^oC. Reaction time was 5 h.

Removing NH_3 from reaction solution: After reaction, the certain volume (5 mL) of solution was taken to remove ammonia with evaporator. Most of ammonia and water was removed after 1.5 h at 100 mbar and 333 K. The solution was then diluted with water to the previous volume (5 mL) with volumetric flask.

Recycle experiments: For recycling experiment, the catalyst was washed with ethanol and DI water after reaction for five times. The washed catalyst was freeze-dried to remove the remaining water overnight for the next usage.

^{1}H NMR experiments: ^{1}H NMR experiment was conducted on a Bruker Ascend 400 MHz NMR spectrometer. For alanine decomposition experiment, the liquid reaction mixture was filtered and analyzed directly. For lactic acid to alanine experiment, the liquid reaction mixture was filtered, freeze-dried and re-dissolved in deuterium oxide (D2O, 99.9 atom %D) for analysis.

Cyclic voltammogram experiments: The cyclic voltammograms of hydroxyl acids and related amino acids were recorded on a CorrTest CS electrochemical workstation. The working electrode was the glassy carbon electrode. In addition, the Pt plate served as the counter electrode, and the saturated calomel electrode served as the reference electrode. A 0.1 M Na_2SO_4 aqueous solution was used as the electrolyte. The scan rate is 50 mV/s. The concentration for hydroxyl acids and amino acids was 0.1 M. Due to the insolubility problem, the concentration of aspartic acid, phenylalanine and phenylglycine was decreased to 0.01M.

Computational setup: The hexagonal wurtzite CdS (100) surface was modeled by a six Cd-S di-layer slab with a 2×2 unit cell, as shown in Supplementary Fig. 12. The structure data of primitive(Lattice constants of a = b = 4.21 Å and c = 6.84 Å.) cell was download from the website of Materials Project.[4] The slabs were separated by a vacuum space of 15 Å. The bottom four layers were fixed during geometry optimization. These illustrations from the computational models were visualized by the VESTA software.[5]

Density functional theory (DFT) calculations were carried out via the Perdew-Burke-Ernzerhof (PBE) exchange-correlation functional[6] as implemented in the Vienna ab initio simulation package (VASP).[7-10] The valence electrons were described by plane wave basis sets with a plane-wave cut-off energy of 400 eV, and the k-points sampling for all structural models was generated following the Monkhorst-Pack procedure with a 2×3×1 mesh.[11] The Grimme's dispersion correction was employed during each geometry optimization step.[12] The transition state (TS) structures were calculated using the climbing-image nudged elastic band (CI-NEB) method[13, 14] and the dimer method.[15] All structures were refined until the Hellman-Feynman forces on each ion were lower than 0.05 eV/Å.

Calculation of bond dissociation energies: Homolytic bond dissociation energies (BDEs) were calculated at the B3LYP/def2TZVP [16, 17] level of theory using Gaussian 16 Rev. A.03.[18] Frequency calculations confirmed that the optimized structures were indeed local minimum structures. Radical fragments were handled with the equivalent UB3LYP functional.

Homolytic BDEs are defined in the following as:

BDE = E (radical fragment A) + E (radical fragment B) – E (A–B)

Calculation of redox potentials: For the calculation of redox potentials of the conversion of lactate (Lac) into alanine (Ami) with pyruvate (Pyr) and the corresponding imine (Imi) as intermediates, the following Born–Haber cycles and equations were used:

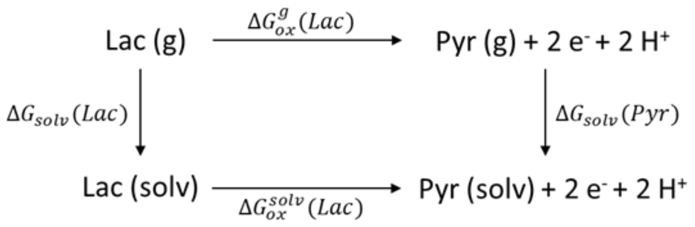

$$\Delta G_{ox}^{solv}(Lac) = \Delta G_{ox}^{g}(Lac) + \Delta G_{solv}(Pyr) - \Delta G_{solv}(Lac)$$

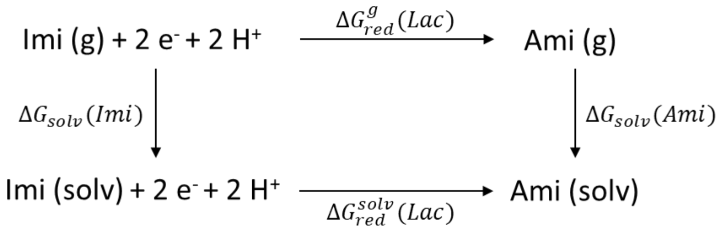

$$\Delta G_{ox}^{solv}(Imi) = \Delta G_{ox}^{g}(Imi) + \Delta G_{solv}(Ami) - \Delta G_{solv}(Imi)$$

Previously determined values for the standard hydrogen electrode [19] and reported values for the proton solvation energy in water were used [20] to calculate the standard electrode potential from the Gibbs free energy change of oxidation and reduction. All calculations were again carried out at the B3LYP/def2TZVP [16, 17] level of theory with Gaussian 16 Rev. A.03.[18] Zero-point energy and thermal corrections were considered from frequency calculations. For the solvation energies in water, the polarizable continuum model (PCM) was employed with an ε value of 78.36.

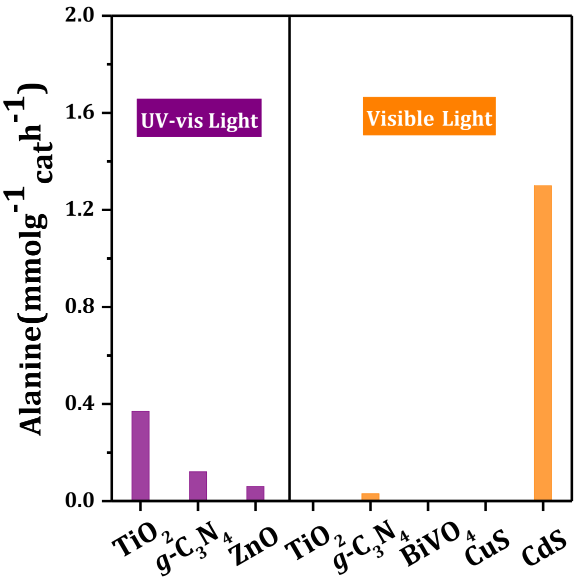

Supplementary Figure 1. Alanine formation rates over different semiconductors under UV-vis or Visible light. Reaction conditions: 10 mg photocatalyst, 20 mmol lactic acid, 4 mL ammonia solution (25 wt%), 16 mL deaerated water, T = 50 ^oC, 1 bar N_2, t = 4 h.

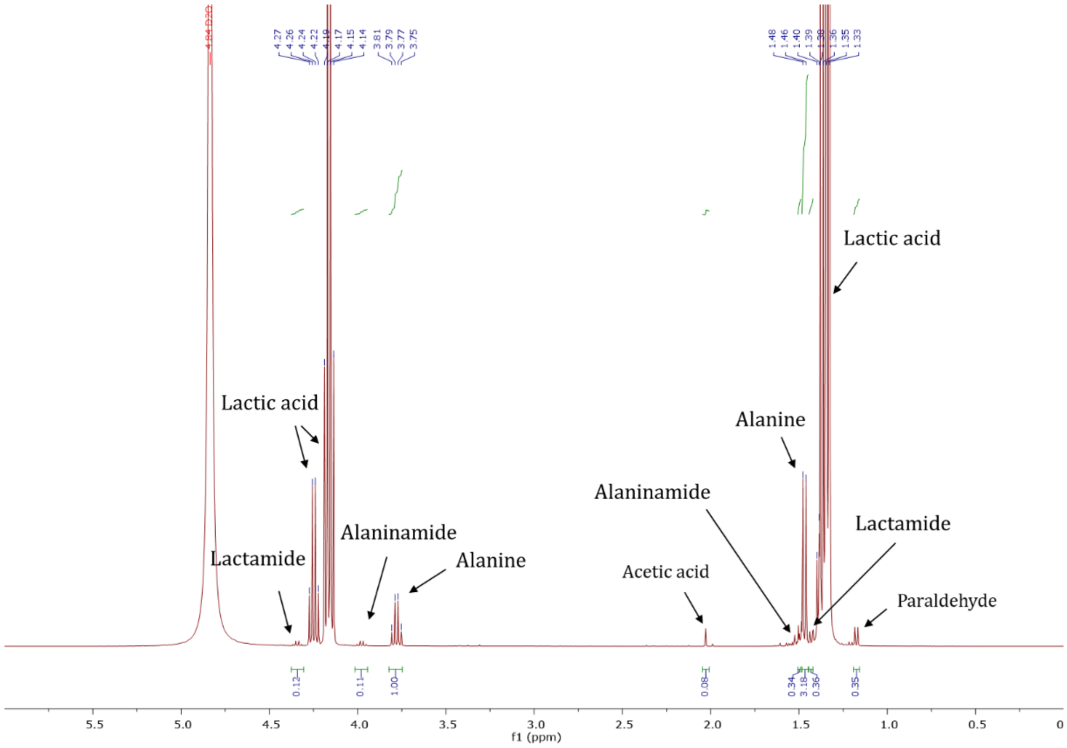

Supplementary Figure 2. ^{1}H NMR spectra of reaction mixture after 10 h. Reaction conditions: 10 mg CdS nanosheet, 20 mmol lactic acid, 4 mL ammonia solution (25 wt%), 16 mL deaerated water, t = 10 h, T = 50 ^oC, 1 bar N_2.

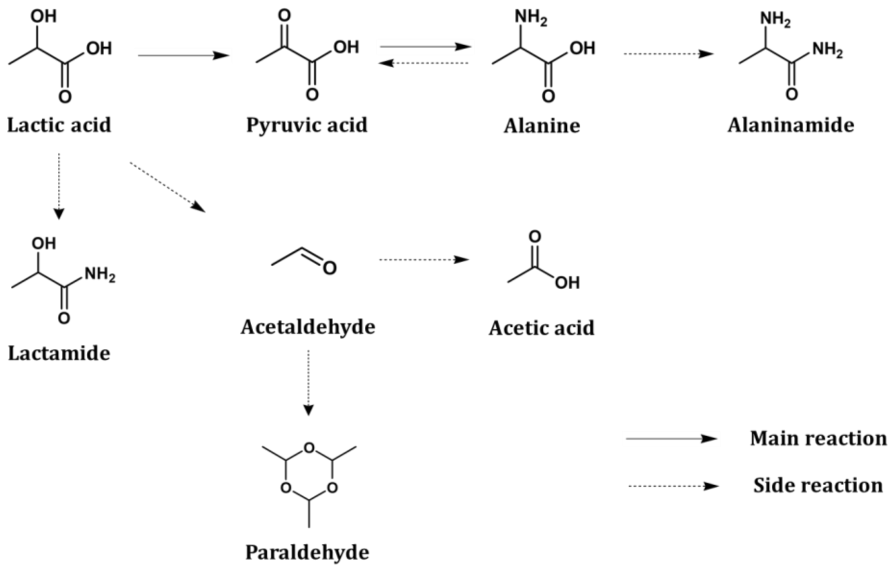

Supplementary Figure 3. Reaction pathway in photocatalysis amination of lactic acid to alanine.

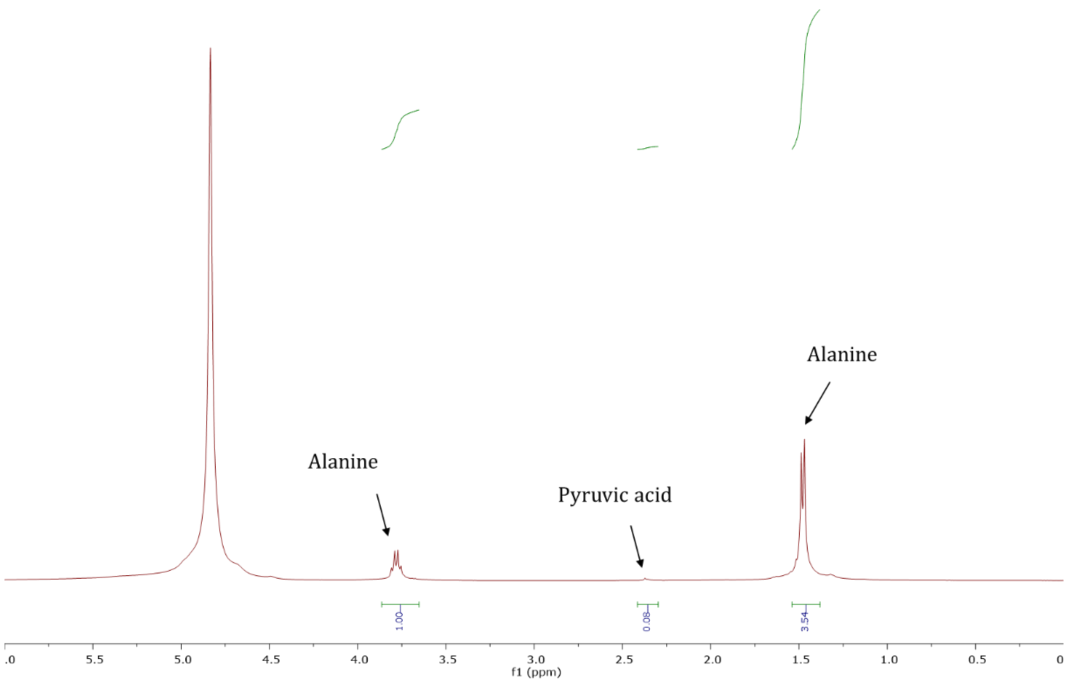

Supplementary Figure 4. ^{1}H NMR spectra of alanine decomposition experiment. Reaction conditions: 2 mg CdS nanosheets, 1 mmol alanine, 2 mL D_2O water, t = 24 h, T = 50 ^oC, 1 bar N_2.

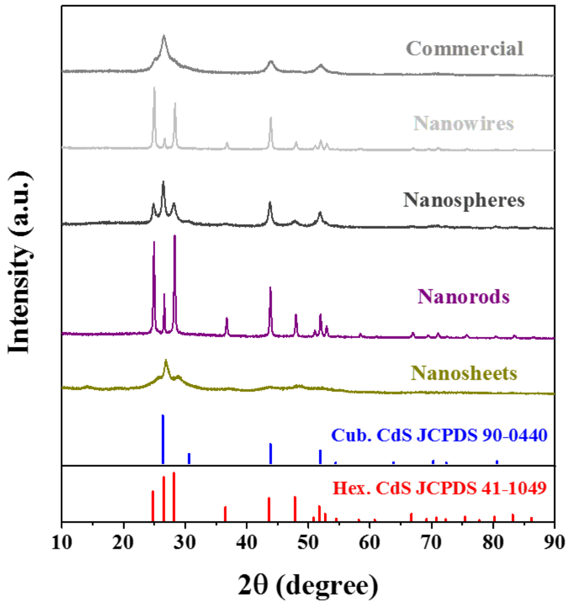

Supplementary Figure 5. XRD spectra for different morphology CdS.

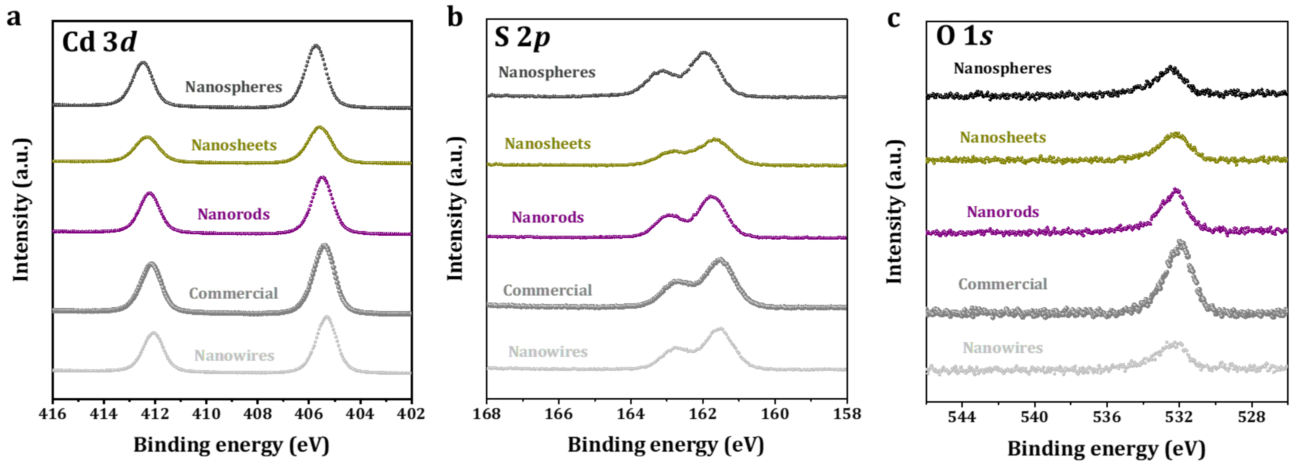

Supplementary Figure 6. XPS spectra for different morphology CdS. **a** Cd 3*d*. **b** S 2*p*. **c** O 1*s*.

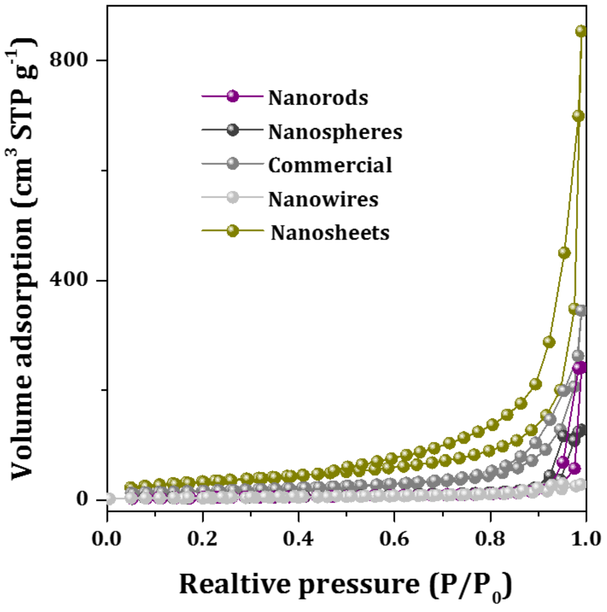

Supplementary Figure 7. N_2 adsorption-desorption isotherms for different morphology CdS.

Supplementary Table 1 BET surface area of different morphology CdS

Sample	BET Surface Area (m^2 g^{-1})
Nanosheets	123.4
Commercial	58.2
Nanospheres	17.3
Nanorods	16.4
Nanowires	12.1

Supplementary Table 2 Alanine yield comparison among different amounts of CdS

Entry	Sample	Alanine yield/mmol [a]	Alanine yield/mmol [b]
1	Nanosheets	0.420	0.420
2	Nanorods	0.096	0.103
3	Nanospheres	0.072	0.075
4	Commercial	0.052	0.058
5	Nanowires	0.012	0.013

[a] Reaction conditions: 10 mg CdS, 20 mmol lactic acid, 4 mL ammonia solution (25 wt%), 16 mL deaerated water,, T = 50 °C, 1 bar N2, visible light, t = 4 h.

[b] Reaction conditions: CdS (commercial 21 mg, nanorods 75 mg, nanospheres 71 mg, nanowires 101 mg or nanosheets 10 mg), 20 mmol lactic acid, 4 mL ammonia solution (25 wt%), 16 mL deaerated water,, T = 50 °C, 1 bar N2, visible light, t = 4 h.

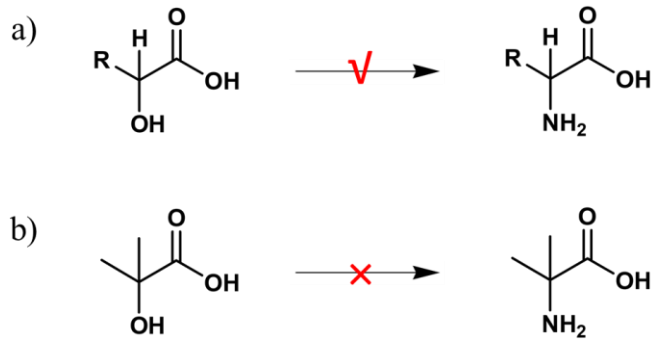

Supplementary Figure 8. The effect of C_{α}-H on photocatalysis amination of α-hydroxyl acids to amino acids. **a** α-hydroxyl acids with C_{α}-H. **b** α-hydroxyl acid without C_{α}-H. Reaction conditions: 10 mg nanosheets, 20 mmol substrates, 4 mL ammonia solution (25 wt%), 16 mL deaerated water, T = 50 ^{o}C, 1 bar N_2, visible light, t = 4 h.

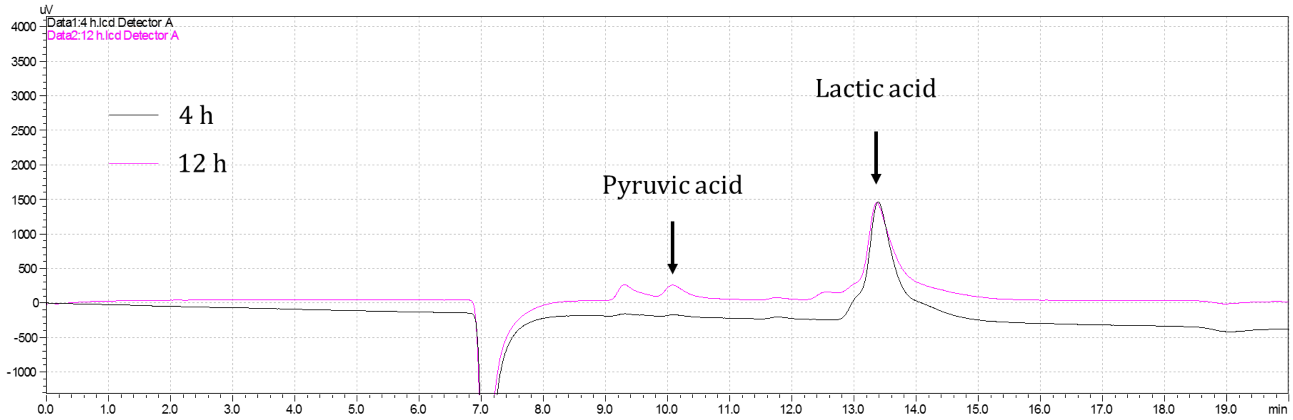

Supplementary Figure 9. HPLC spectra (RI detector) of lactic acid conversion as a function of reaction time. Reaction conditions: 10 mg nanosheets, 20 mmol lactic acid, 20 mL deaerated water, T = 50 ^{o}C, 1 bar N_2, visible light.

Supplementary Table 3 Catalytic performances with pyruvic acid as substrates over CdS

Entry	Substrate	Catalyst	Alanine formation rate (mmol g_{cat}^{-1} h^{-1})
1	Pyruvic acid	Nanosheets	1.8
2	Pyruvic acid	Nanorods	1.9
3	Pyruvic acid	Nanospheres	2.4
4	Pyruvic acid	Nanowires	1.9
5	Pyruvic acid	Commercial	2.2

Reaction conditions: 10 mg CdS, 20 mmol pyruvic acid, 4 mL ammonia solution (25 wt%), 16 mL deaerated water, T = 50 ^{o}C, 1 bar N_2, visible light, t = 4 h.

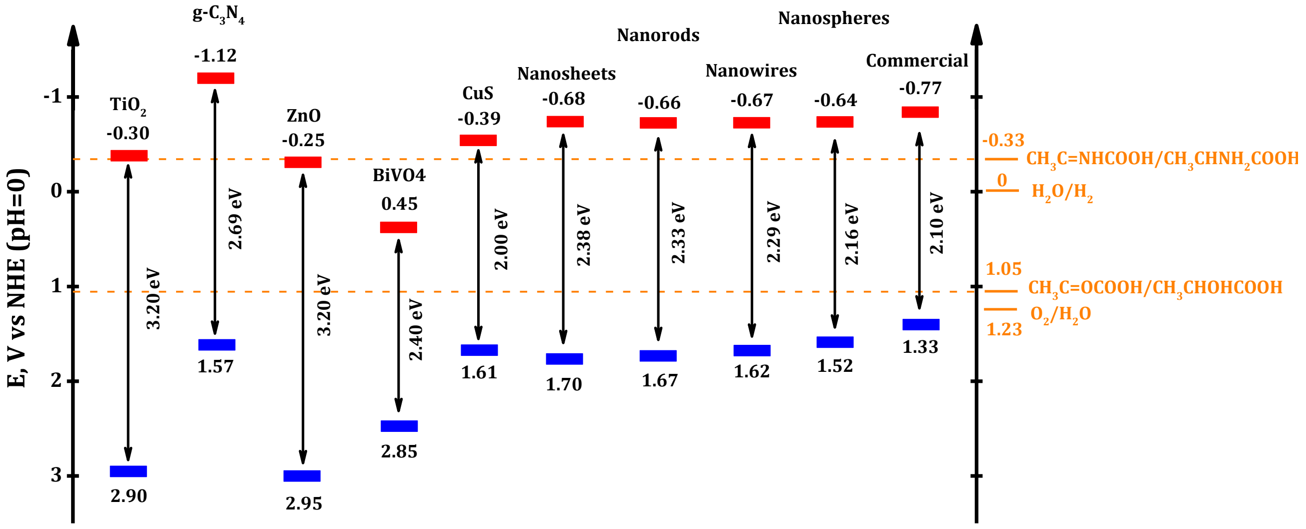

Supplementary Figure 10. Redox potentials of related reactions and conduction band and valence band positions of selected semiconductors. The conduction band potential E_{CB} was very close to the flat band potentials for n-type semiconductors (Supplementary Figure 8 and Supplementary Table 5). The valence band potential E_{VB} was calculated according to the E_{BG} and E_{CB}. The positions of E_{CB} and E_{CB} for TiO_2, *g*-C_3N_4, ZnO, $BiVO_4$ and CuS were collected from the references.[21-23]

It seems that the photocatalytic amination of lactic acid to alanine can only proceed when the conduction band (EVB) position of photocatalyst is higher than the imino acid/alanine redox potential (-0.33 V) and the valence band (ECB) position is lower than the lactic acid/pyruvic acid redox potential (1.05 V). This criterial matches well with experimental observation (Supplementary Fig. 1): $BiVO_4$ is inactive for the desired transformation since the band gap does not fall within desired value. On the other hand, TiO_2, ZnO, g-C_3N_4 and various CdS materials are active for the reaction. CuS was an exception, possibly due to its poor adsorption of substrate and quick recombination of the charge carriers as indicated in the literature.[24, 25]

Supplementary Table 4 Redox potentials of related reactions

Reaction steps	Redox potential [a] vs. NHE [b] (pH = 7) (V)	Redox potential vs. NHE (pH = 0) (V)
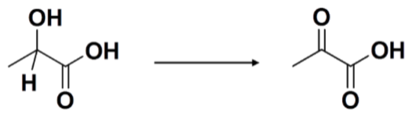	0.64 (1.10)[c]	1.05
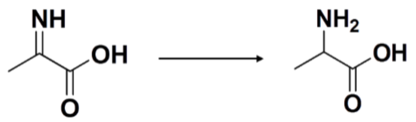	-0.74	-0.33

[a] Redox potentials were calculated based on B3LYP/def2TZVP model;
[b] NHE means the normal hydrogen electrode;
[c] Experiment data: 1.1 V vs. SCE at a glassy carbon electrode in 0.5 M K_2SO_4 solution.[26] SCE means the saturated calomel electrode.

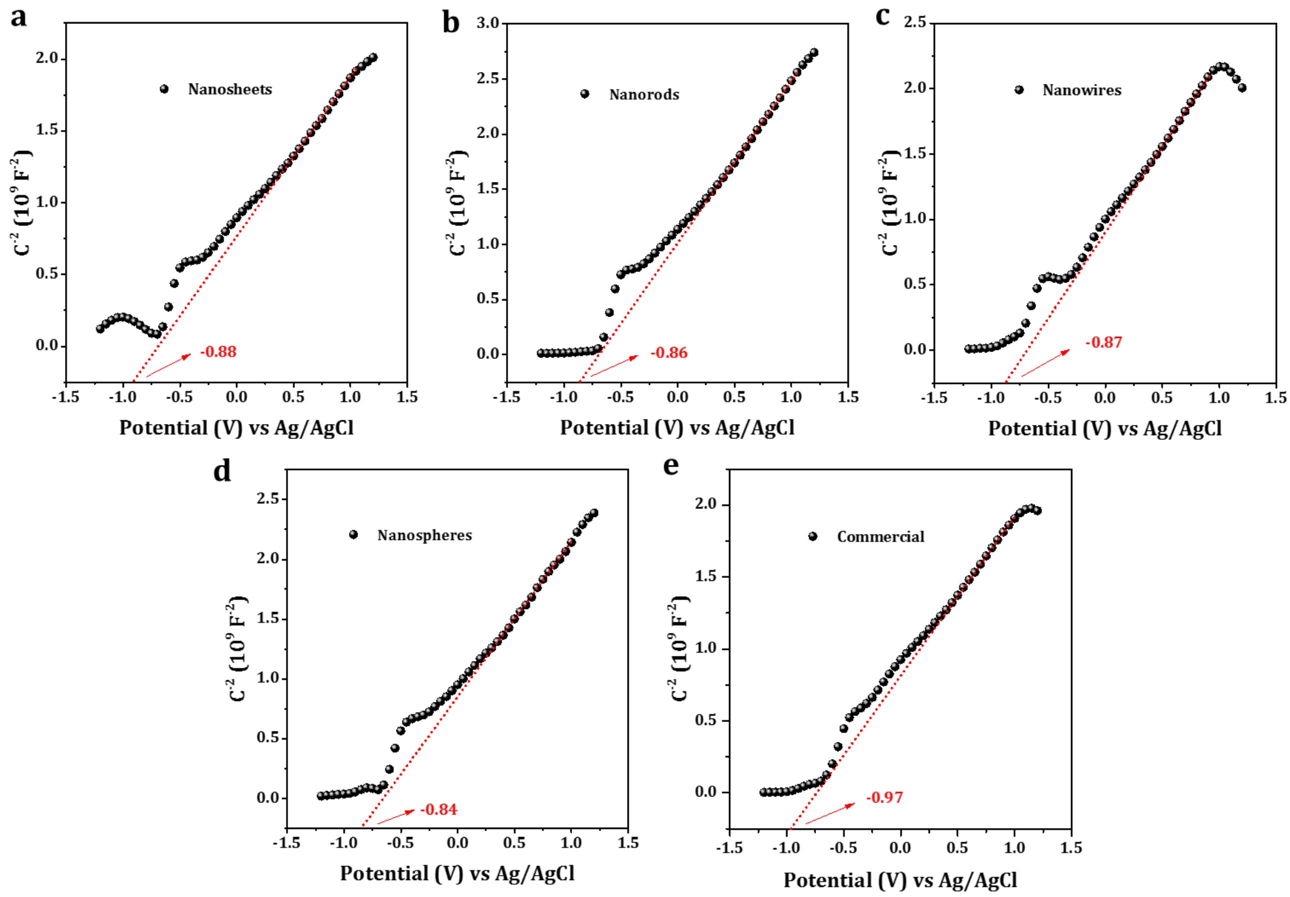

Supplementary Figure 11. Mott–Schottky plots for different morphology CdS in 0.2 M Na_2SO_4 aqueous solution (pH = 6.8). **a** CdS nanosheets. **b** CdS nanorods. **c** CdS nanowires. **d** CdS nanospheres. **e** Commercial CdS.

Supplementary Table 5 Flat band potentials of different morphology CdS at NHE [a] (pH=0)

Samples	Flat band potentials vs. Ag/AgCl (pH = 6.8) (V)	Flat band potentials [b] vs. NHE (pH = 0) (V)
Nanosheets	-0.88	-0.68
Nanorods	-0.86	-0.66
Nanowires	-0.87	-0.67
Nanospheres	-0.84	-0.64
Commercial	-0.97	-0.77

[a] NHE means the normal hydrogen electrode.

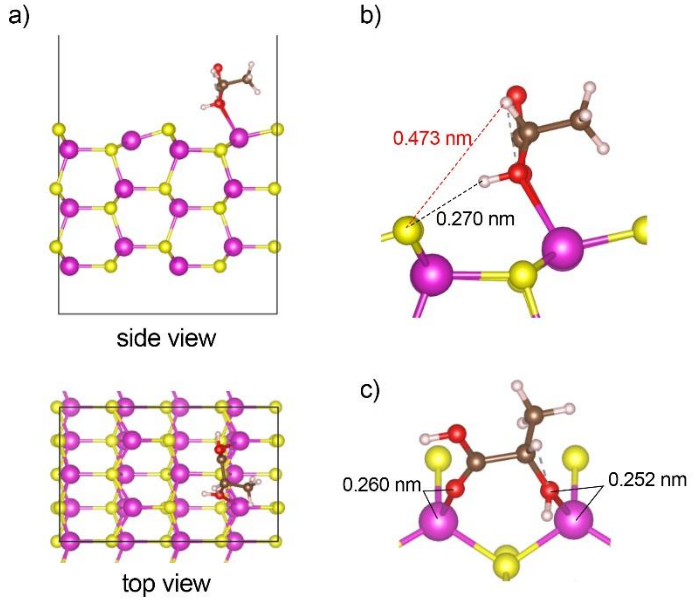

Supplementary Figure 12. The optimized structures of the adsorption lactic acid on CdS (100) surface. **a** Top views and side view. **b** The distances between the H atoms and the S site. **c** The distances between the absorbed O atoms and the Cd sites.

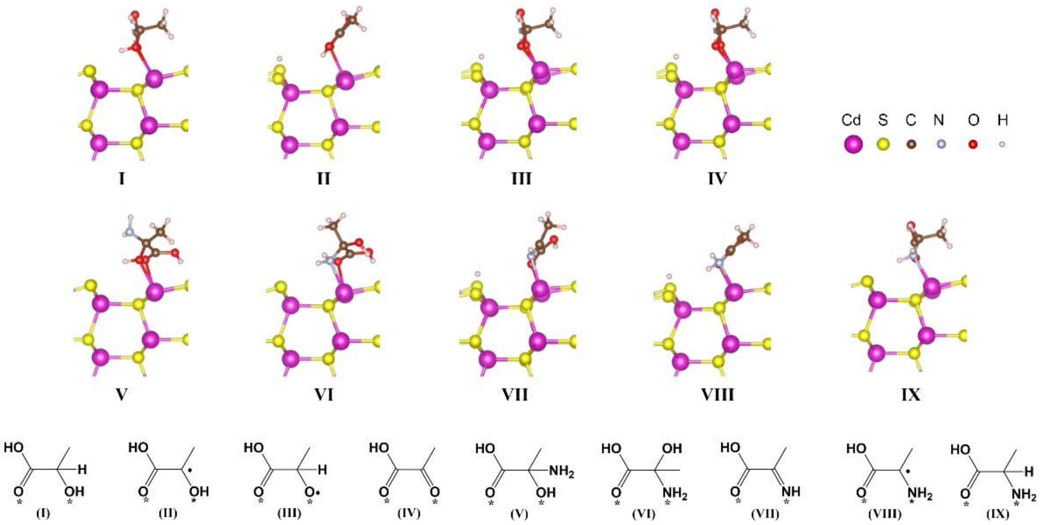

Supplementary Figure 13. DFT-optimized structures on CdS (100) surface. Cd, S, C, N, O and H atoms are represented in purple, yellow, brown, gray, red, and white, respectively.

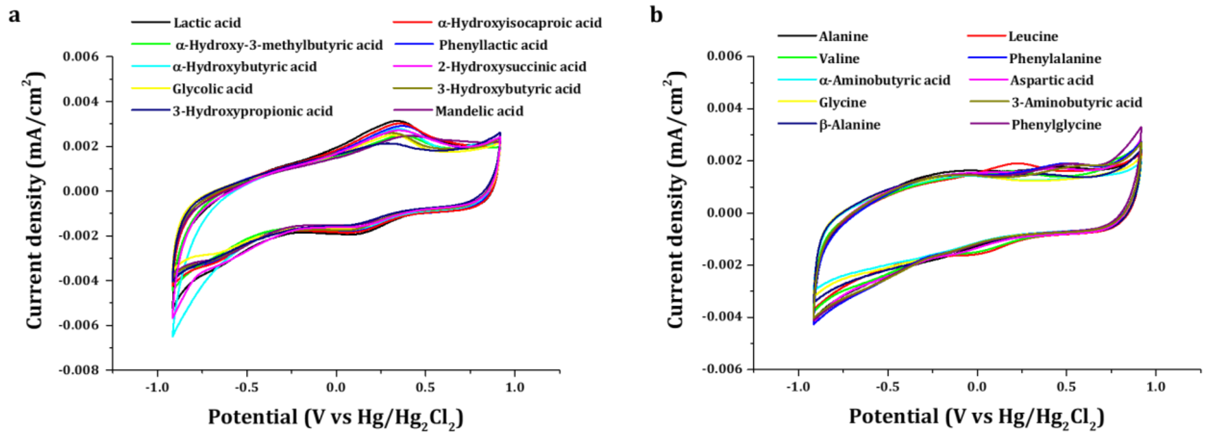

Supplementary Figure 14. The cyclic voltammograms of various hydroxyl acids and corresponding amino acids. **a** hydroxyl acids. **b** amino acids.

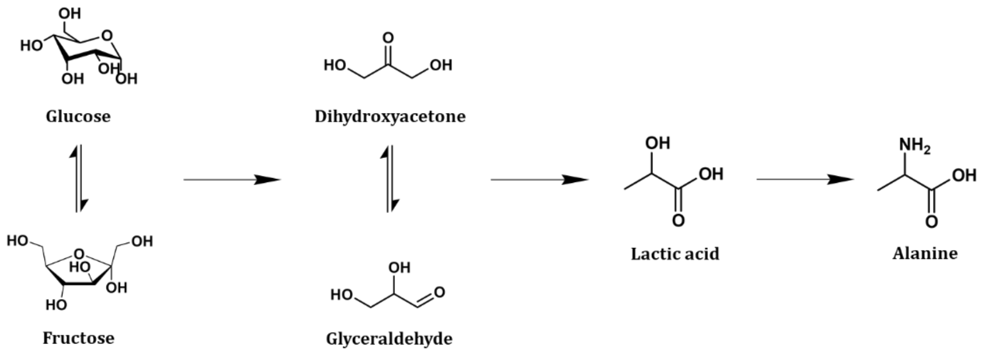

Supplementary Figure 15. Reaction scheme for photocatalytic amination of glucose to alanine.

Supplementary Table 6 Photocatalytic conversion of glucose to alanine over CdS.

Entry	Photo catalyst	Alkaline	Conc./ M	Con. /%	Lactic acid/mmol	Alanine/ mmol	Alanine formation rate/ mmol g_{cat}^{-1} h^{-1}
1	Nanosheets	-	-	73.6	0	0	0
2	Nanosheets	NaOH	0.20	97.5	0.48	0.021	0.26
3	Nanosheets	KOH	0.20	97.0	0.36	0.017	0.21
4	Nanosheets	LiOH	0.20	97.3	0.27	0.015	0.19
5	Nanosheets	$Ba(OH)_2$	0.10	99.5	1.16	0.008	0.10
6	Nanosheets	NaOH	0.30	98.3	0.92	0.027	0.34
7	Nanosheets	NaOH	0.50	99.0	1.48	0.019	0.24
8	Nanorods	NaOH	0.30	98.1	0.94	0.023	0.11
9	Commercial	NaOH	0.30	98.6	0.90	0.022	0.08
10	Nanowires	NaOH	0.30	99.1	0.91	0.025	0.07
11	Nanospheres	NaOH	0.30	97.1	0.89	0.023	0.08

Reaction conditions: CdS 10 mg, glucose 2 mmol, 4 mL ammonia solution (25 wt%), 16 mL deaerated water, stir = 600 rpm, N_2 1 bar, time = 8 h.

Supplementary Table 7 The effect of base concentration on photocatalytic conversion of lactic acid to alanine over CdS nanosheets.

Entry	Base	Concentration/M	Alanine/mmol	Alanine Formation rate/ mmol g_{cat}^{-1} h^{-1}
1	-	-	0.151	1.89
2	NaOH	0.20	0.029	0.36
3	NaOH	0.30	0.016	0.20
4	NaOH	0.50	0.012	0.15

Reaction conditions: nanosheets 10 mg, lactic acid 1 mmol, 4 mL ammonia solution (25 wt%), 16 mL deaerated water, stir=600 rpm, N_2 1 bar, time = 8 h.

Supplementary References

1. Liu, J., Zhang, T., Wang, Z., Dawson, G. & Chen, W. Simple pyrolysis of urea into graphitic carbon nitride with recyclable adsorption and photocatalytic activity. *J. Mater. Chem.* **21**, 14398-14401 (2011).
2. Li, R. *et al.* Spatial separation of photogenerated electrons and holes among {010} and {110} crystal facets of $BiVO_4$. *Nat. Commun.* **4**, 1432 (2013).
3. Zhang, Y. *et al.* Biomolecule-assisted, environmentally friendly, one-pot synthesis of CuS/reduced graphene oxide nanocomposites with enhanced photocatalytic performance. *Langmuir* **28**, 12893-12900 (2012).
4. Persson, K. Materials Data on CdS (SG:186) by Materials Project. *United States* (2014).
5. Momma, K. & Izumi, F. VESTA 3 for three-dimensional visualization of crystal, volumetric and morphology data. *J. Appl. Crystallogr.* **44**, 1272-1276 (2011).
6. Perdew, J. P., Burke, K. & Ernzerhof, M. Generalized gradient approximation made simple. *Phys. Rev. Lett.* **77**, 3865 (1996).
7. Kresse, G. & Furthmüller, J. Efficiency of ab-initio total energy calculations for metals and semiconductors using a plane-wave basis set. *Comput. Mater. Sci.* **6**, 15-50 (1996).
8. Kresse, G. & Furthmüller, J. Efficient iterative schemes for ab initio total-energy calculations using a plane-wave basis set. *Phys. Rev. B* **54**, 11169 (1996).
9. Kresse, G. & Hafner, J. Ab initio molecular dynamics for open-shell transition metals. *Phys. Rev. B* **48**, 13115 (1993).
10. Kresse, G. & Hafner, J. Ab initio molecular-dynamics simulation of the liquid-metal–amorphous-semiconductor transition in germanium. *Phys. Rev. B* **49**, 14251 (1994).
11. Monkhorst, H. J. & Pack, J. D. Special points for Brillouin-zone integrations. *Phys. Rev. B* **13**, 5188 (1976).
12. Grimme, S., Antony, J., Ehrlich, S. & Krieg, H. A consistent and accurate ab initio parametrization of density functional dispersion correction (DFT-D) for the 94 elements H-Pu. *J. Chem. Phys.* **132**, 154104 (2010).
13. Henkelman, G. & Jónsson, H. Improved tangent estimate in the nudged elastic band method for finding minimum energy paths and saddle points. *J. Chem. Phys.* **113**, 9978-9985 (2000).
14. Krukau, A. V., Vydrov, O. A., Izmaylov, A. F. & Scuseria, G. E. Influence of the exchange screening parameter on the performance of screened hybrid functionals. *J. Chem. Phys.* **125**, 224106 (2006).
15. Henkelman, G. & Jónsson, H. A dimer method for finding saddle points on high dimensional potential surfaces using only first derivatives. *J. Chem. Phys.* **111**, 7010-7022 (1999).
16. Stephens, P. J., Devlin, F., Chabalowski, C. & Frisch, M. J. Ab initio calculation of vibrational absorption and circular dichroism spectra using density functional force fields. *J. Phys. Chem.* **98**, 11623-11627 (1994).
17. Weigend, F. & Ahlrichs, R. Balanced basis sets of split valence, triple zeta valence and quadruple zeta valence quality for H to Rn: Design and assessment of accuracy. *Phys. Chem. Chem. Phys.* **7**, 3297-3305 (2005).
18. Frisch, M. J. et al. Gaussian 16, Revision A.03 (Gaussian, Inc., Wallingford CT, 2016).
19. Tripkovic, V., Björketun, M. E., Skúlason, E. & Rossmeisl, J. Standard hydrogen electrode and potential of zero charge in density functional calculations. *Phys. Rev. B* **84**, 115452 (2011).
20. Marković, Z., Tošović, J., Milenković, D. & Marković, S. Revisiting the solvation enthalpies and free energies of the proton and electron in various solvents. *Comput. Theor. Chem.* **1077**, 11-17 (2016).
21. Tamirat, A. G., Rick, J., Dubale, A. A., Su, W. & Hwang, B. Using hematite for photoelectrochemical water splitting: a review of current progress and challenges. *Nanoscale Horiz.* **1**, 243-267 (2016).
22. Lu, Q., Yu, Y., Ma, Q., Chen, B. & Zhang, H. 2D transition-metal-dichalcogenide-nanosheet-based composites for photocatalytic and electrocatalytic hydrogen evolution reactions. *Adv. Mater.* **28**, 1917-1933 (2016).
23. Xie, S. *et al.* Visible light-driven C-H activation and C-C coupling of methanol into ethylene glycol. *Nat. Commun.* **9**, 1181 (2018).

24. Wu, J. *et al.* CuS/RGO hybrid photocatalyst for full solar spectrum photoreduction from UV/Vis to near-infrared light. *J. Colloid Interface Sci.* **517**, 80-85 (2018).
25. Lai, C. *et al.* Fabrication of CuS/$BiVO_4$ (040) binary heterojunction photocatalysts with enhanced photocatalytic activity for Ciprofloxacin degradation and mechanism insight. *Chem. Eng. J.* **358**, 891-902 (2019).
26. Harada, H., Sakata, T. & Ueda, T. Effect of semiconductor on photocatalytic decomposition of lactic acid. *J. Am. Chem. Soc.* **107**, 1773-1774 (1985).
